# The Complete Mitogenome of *Apostasia fujianica* Y.Li & S.Lan and Comparative Analysis of Mitogenomes across Orchidaceae

**DOI:** 10.3390/ijms25158151

**Published:** 2024-07-26

**Authors:** Qinyao Zheng, Xiaoting Luo, Ye Huang, Shi-Jie Ke, Zhong-Jian Liu

**Affiliations:** 1 Key Laboratory of National Forestry and Grassland Administration for Orchid Conservation and Utilization at Landscape Architecture and Arts, Fujian Agriculture and Forestry University, Fuzhou 350002, China; 2 College of Forestry, Fujian Agriculture and Forestry University, Fuzhou 350002, China

**Keywords:** Apostasiaceae, monocots, phylogenetic analysis

## Abstract

*Apostasia fujianica* belongs to the genus *Apostasia* and is part of the basal lineage in the phylogenetic tree of the Orchidaceae. Currently, there are only ten reported complete mitochondrial genomes in orchids, which greatly hinders the understanding of mitochondrial evolution in Orchidaceae. Therefore, we assembled and annotated the mitochondrial genome of *A. fujianica*, which has a length of 573,612 bp and a GC content of 44.5%. We annotated a total of 44 genes, including 30 protein-coding genes, 12 tRNA genes, and two rRNA genes. We also performed relative synonymous codon usage (RSCU) analysis, repeat sequence analysis, intergenomic transfer (IGT) analysis, and Ka/Ks analysis for *A. fujianica* and conducted RNA editing site analysis on the mitochondrial genomes of eight orchid species. We found that most protein-coding genes are under purifying selection, but *nad6* is under positive selection, with a Ka/Ks value of 1.35. During the IGT event in *A. fujianica*’s mitogenome, the *trnN*-*GUU*, *trnD*-*GUC*, *trnW*-*CCA*, *trnP*-*UGG*, and *psaJ* genes were identified as having transferred from the plastid to the mitochondrion. Compared to other monocots, the family Orchidaceae appears to have lost the *rpl10*, *rpl14*, *sdh3*, and *sdh4* genes. Additionally, to further elucidate the evolutionary relationships among monocots, we constructed a phylogenetic tree based on the complete mitogenomes of monocots. Our study results provide valuable data on the mitogenome of *A. fujianica* and lay the groundwork for future research on genetic variation, evolutionary relationships, and breeding of Orchidaceae.

## 1. Introduction

Mitochondria are essential organelles within cells, responsible for generating adenosine triphosphate (ATP) through oxidative phosphorylation, regarded as the cell’s powerhouse [1,2]. Unlike the conserved animal mitochondrial genomes, there exist significant differences in both size and structure in the currently assembled plant mitochondrial genomes. For example, the smallest reported green plant mitochondrial genome, that of *Polytomella capuana*, is only 13 kb [3], while large mitochondrial genomes can reach up to 1600 Mb in muskmelon [4]. Notably, variations in the mitogenome size also occur within the same plant genus, as observed in *Citrullus* and *Silene* species [5]. Additionally, plant mitochondria exhibit diverse morphologies, including linear, fragmented, circular, branched, and multi-chromosomal structures [5]. In general, most mitogenomes of angiosperms are arranged in a single master circle, containing a set of intact genes and numerous subcircles. This multi-chromosomal structure has been observed in various plants genera, such as *Amborella*, *Cucumis*, *Lophophytum*, *Saccharum*, and *Silene* [6]. This complexity poses significant difficulties and challenges for mitochondrial assembly.

Mitochondrial genes are generally conservative in both type and quantity, including protein-coding genes (PCGs), ribosomal RNA (rRNA), and transfer RNA (tRNA). Over evolutionary time, the number of mitochondrial genes has reduced from an estimated thousands to only a few dozen in most eukaryotes [7]. Among extant higher plants, the number of mitochondrial protein-coding genes ranges from 24 to 42 [8]. Additionally, compared to nuclear and plastid markers, mitochondrial genomes are rarely used to establish the phylogenetic relationships. However, mitogenomes hold immense potential in resolving phylogenetic relationships for species where they have been successfully employed [9]. For example, in Orchidaceae, phylogenetic trees constructed using mitochondrial DNA (mtDNA), in comparison to those built using plastid genes, show a reversal in the positions of Cypripedioideae and Vanilloideae. This reversal better aligns with hypotheses regarding orchid anther evolution, which suggest a progression from three fertile anthers to one degenerate anther, two fertile anthers, and finally a single fertile anther). In addition, these mtDNA-based analyses have resolved the phylogenetic positions of Gastrodieae, Epipogiinae, and *Risleya* [10].

The Orchidaceae family is one of the largest families of flowering plants, comprising approximately 29,500 species [11]. Currently, there are fewer than 50 complete mitochondrial genomes of monocotyledonous plants available on the NCBI platform, with orchids accounting for 10 of them [12]. Among them, *Apostasia fujianica* [13] belongs to the basal group of the Orchidaceae phylogenetic tree [14,15], Apostasiaceae, and represents one of the most primitive orchids [16], with its mitochondrial genome capable of providing insights into the characteristics of ancient orchid mitochondria. Here, we generated a newly completed mitochondrial genome of *A. fujianica* and conducted a comparative analysis of orchid mitochondrial genomes, including codon analysis, Ka/Ks value analysis, repeat sequence analysis, analysis of intracellular gene transfer (IGT) events, and the establishment of a monocotyledonous ML phylogenetic tree based on the full length of mitochondrial genomes. We believe that our results can serve as valuable molecular resources for future research on genetic variation and systematic evolution of the orchid family.

## 2. Results

### 2.1. Mitogenome Characteristics

**Figure 1:**
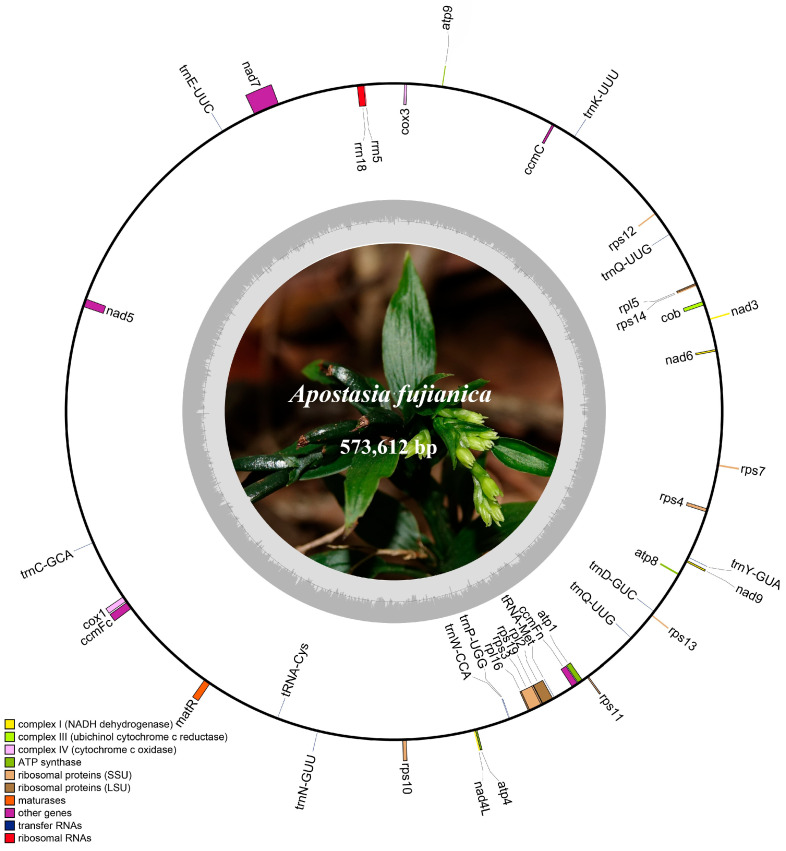
Mitochondrial annotation map of *A. fujianica*. The dark gray region represents GC content, while the light gray region represents AT content.

Through de novo assembly, the mitogenome size of *A. fujianica* was determined to be 573,612 bp, consisting of 25 contigs, and a circular structure was manually depicted (Figure 1). The GC content was measured to be 44.5%. In total, 44 mitochondrial genes were annotated, comprising 30 coding sequences (CDS), 12 tRNAs, and two rRNAs. The lengths of tRNAs ranged from 71 bp (*trnC*-*GCA*) to 83 bp (*trnY*-*GUA*), collectively representing 0.15% of the total length. The rRNAs consisted of *rrn18* (1994 bp) and *rrn5* (117 bp), collectively accounting for 0.37% of the total length. The longest CDS was *matR*, measuring 1926 bp, while the shortest was *psaJ*, measuring 138 bp. From Figure 1, it can be observed that the CDS were categorized into eight different classes, with ribosomal proteins (SSU) containing the highest number of genes (nine), followed by complex I (NADH dehydrogenase), with six genes. Complex III (ubichinol cytochrome c reductase) and maturases had the fewest genes, each with one (*cob* and *matR*, respectively).

### 2.2. Codon Preference Analysis

**Figure 2:**
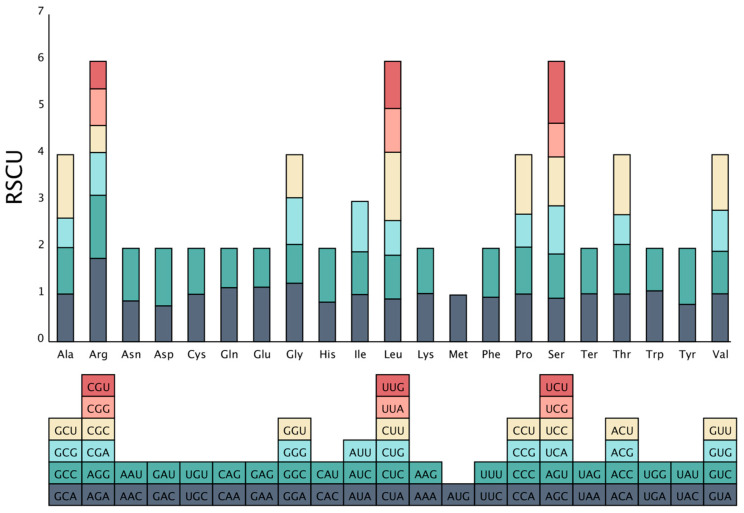
Relative synonymous codon usage (RSCU) in *A. fujianica* mitogenome.

As depicted in Figure 2, 21 amino acids and 64 codons were identified from *A. fujianica*’s mitogenome. The results indicate that 32 codons had the relative synonymous codon usage (RSCU) values greater than 1, while 31 codons had values less than 1. Notably, the codon AUG, encoding Methionine (Met), had a unique RSCU value of 1. Among these, the codon AGA, encoding Arginine (Arg), had the highest RSCU value of 1.78, followed by CUU, encoding Leucine (Leu), with an RSCU value of 1.46, while the lowest value was observed for the codon CGC, encoding Arginine (Arg), with an RSCU value of 0.58. Additionally, among the six codons encoding Arg, it is evident that the codons AGA and AGG were utilized more frequently than the other four codons. For Leu, CUU exhibited the highest frequency of usage, and for Serine (Ser), UCU had the highest RSCU value.

### 2.3. Repeat Sequence and Ka/Ks Analysis

**Figure 3:**
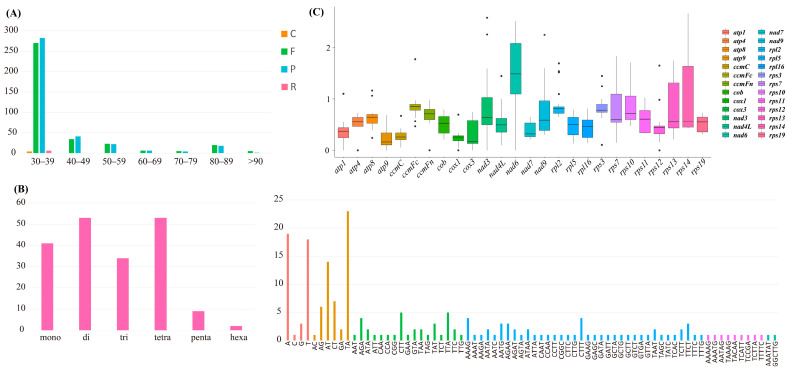
(**A**) Long repeats in *A. fujianica* mitogenome. (**B**) SSR distribution in *A. fujianica* mitogenome. (**C**) Ka/Ks values of 26 PCGs from eight Orchidaceae mitogenomes.

As shown in Figure 3A, a total of 747 long repeats were identified. Among them, the long repeats found in the 30–39 bp range (562 long repeats) far exceed those in the other six ranges. Specifically, the two types forward (F) and palindrome (P) constituted the majority, with quantities of 270 and 282, respectively. In contrast, there are only four and six repeats of types complement (C) and reverse (R), respectively. It is worth noting that types C and R were identified exclusively in the 30–39 range. Additionally, in the ranges 40–49/50–59/80–89, there were 75/45/38-long repeats, respectively, while in the ranges 60–69/70–79/>90, there were 12/9/6, respectively. To identify simple sequence repeats (SSRs) in the mitogenome of *A. fujianica*, we identified a total of 192 SSRs (Figure 3B). Among them, di- and tetra-nucleotide SSRs were the most abundant, totaling 53 each. Following these were mono- (41) and tri-nucleotide (34) SSRs. The numbers of penta- and hexa-nucleotide SSRs were nine and two, respectively. In mononucleotide repeats, the repeat count of A (19) and T (18) was notably higher than that of C (1) and G (3). In dinucleotide repeats, the AT and TA repeats were predominant, with 14 and 23, respectively, while the AC and GA repeats were low, with one and two repeats, respectively. In trinucleotide repeats, the highest repeats were CTT and TTA, both with five repeats, followed by AGA (four repeats). In tetranucleotide repeats, the highest repeats were AAAG and CTTT, both with four repeats.

To analyze the substitution rates of mitochondrial genes in *A. fujianica*’s mitogenome, we selected 26 shared protein-coding genes (PCGs) from eight orchid mitogenomes of *A. fujianica*, *A. shenzhenica*, *Dendrobium wilsonii*, *D. henanense*, *Gastrodia pubilabiata*, *G. elata*, *Phalaenopsis aphrodite*, and *Paphiopedilum micranthum* to calculate the Ka, Ks, and Ka/Ks values. As observed in Figure 3C, the median Ka/Ks values of most PCGs were less than 1, while the *nad6* gene stood out with a median value of 1.35. The *rpl2* gene generated three large outliers of 1.21, 2.63, and 3.53, respectively, while the *rps14* gene also produced a notable outlier of 3.19. Furthermore, the *cox3*/*nad6*/*nad9*/*rps7*/*rps13*/*rps14* genes displayed considerable and discrete degrees of variation, indicating significant variation in the substitution rates of these genes in orchids.

### 2.4. Gene Transfer between Organelle Genomes

**Figure 4:**
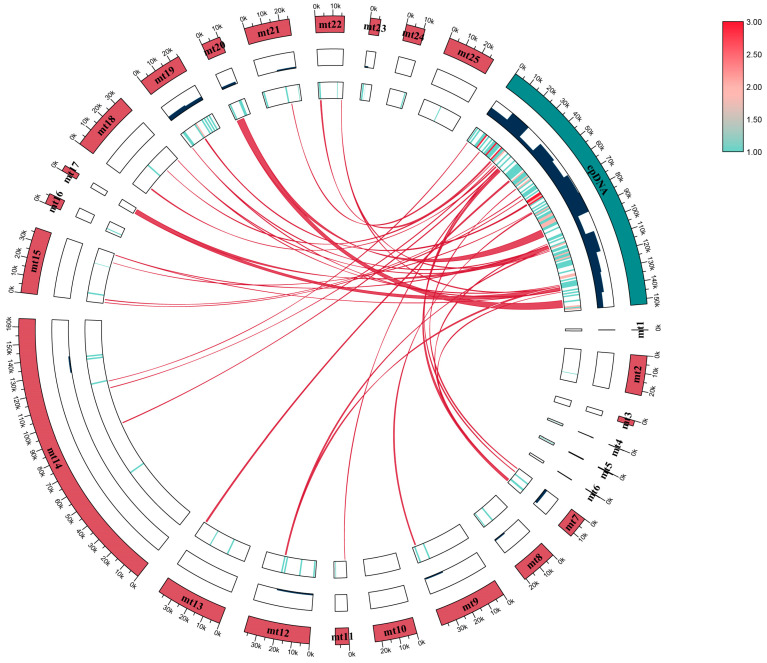
Gene transfer between the mitochondrial and plastid genomes of *A. fujianica*. The outer circle represents the names and lengths of mitochondrial contigs and plastids. The middle circle indicates gene density, while the innermost circle displays the distribution of genes.

To identify inter-organelle gene horizontal transfer in *A. fujianica*, we conducted collinearity analysis between the mitochondrial genome (573,612 bp) and the chloroplast genome (153,179 bp). A total of 41 collinear fragments were identified, ranging from 116 to 164,718 bp, with the GC content varying between 37.5% and 49.8% (Figure 4). Our results indicate the presence of a small number of genes undergoing transfer from the chloroplast to the mitochondrial genome in *A. fujianica*, including four tRNA genes (*trnN*-*GUU*, *trnD*-*GUC*, *trnW*-*CCA*, and *trnP*-*UGG*), as well as *psaJ* (Supplement Table S1). Additionally, it was evident from Table 1 and Figure 4 that the gene density of the mt19 contig was the highest, encompassing nine mitochondrial genes, whereas contig mt1/3/6/10/11/17 did not contain any genes.

### 2.5. RNA Editing Site Prediction and Phylogenetic Analysis

**Figure 5:**
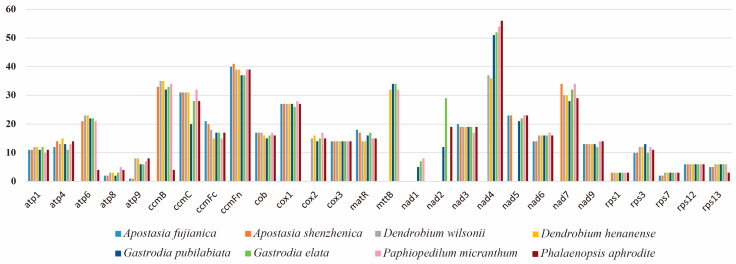
RNA editing sites in eight orchid species.

To identify RNA editing sites, we selected the mitochondrial genomes of eight orchid species for prediction (Figure 5). The number of RNA editing sites in the *nad4* gene of orchid species is remarkably high, typically around 50 sites. This is followed by the *ccmFc* gene, which has about 40 sites. In contrast, the number of editing sites in the *atp8*, *atp9*, *nad1*, *rps1*, *rps7*, *rps12*, and *rps13* genes does not exceed ten. It is noteworthy that the *cox3* and *rps12* genes exhibited consistent RNA editing site counts across all eight Orchidaceae species, with 14 and 6 sites, respectively.

**Figure 6:**
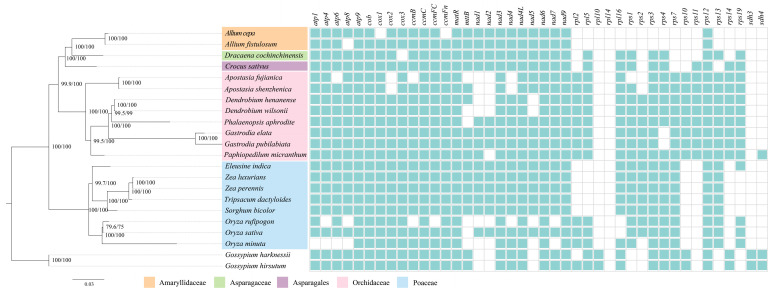
ML phylogenetic tree of monocots based on complete mitochondrial genomes and gene loss of 42 PCGs across species. Green indicates gene presence, while white indicates gene loss.

The phylogenetic analysis showed that the bootstrap values of all nodes exceeded 80% (Figure 6). Orchidaceae emerged as a strongly supported monophyletic group, with node support rates of 100%. It was evident that the subfamily Apostasioideae, containing *A. fujianica*, formed a sister group with other orchid species. Following this, the subfamily Cypripedioideae, represented by *P. micranthum*, established a sister relationship with five species from Epidendroideae. Additionally, we found that all monocotyledonous plants have lost the *rpl10* and *sdh3* genes, while nine genes were shared among monocot species (*atp9*/*cob*/*ccmC*/*matR*/*nad3*/*nad4L*/*nad6*/*nad9*/*rps12*). In Amaryllidaceae, the gene loss is particularly significant, with a total loss of 17 genes. Within Orchidaceae, the *rpl10* and *rpl14* genes were not detected in this family. Among them, both *Apostasia* and *Dendrobium* genera lack the *nad1* and *nad2* genes. Poaceae exhibited a complete loss of *rps10*/*11*/*14* genes.

Moreover, Table 2 reveals a notable diversity in the mitochondrial genome characteristics among eight orchid species. The genome sizes varies from 447,368 bp (*P. micranthum*) to 1,339,825 bp (*G. elata*), with the GC content ranging from 42.1% (*G. pubilabiata*) to 44.6% (*G. elata*). The number of genes also exhibits considerable disparity, spanning from 44 genes in *A. fujianica* to 81 genes in *P. micranthum*. *Dendrobium* species exhibit two or three times the number of tRNA genes compared to other orchid species, whereas *Paphiopedilum* species possess the highest count of protein-coding genes. Additionally, it is worth noting that *Apostasia* species have one fewer rRNA gene compared to other orchid plants.

## 3. Discussion

Mitochondria are present in nearly all eukaryotic cells, serving as the primary ATP supplier for various metabolic pathways and substance synthesis, playing an irreplaceable role in maintaining the normal physiological functions of organisms [17]. Compared to animal mitochondrial genomes, plant mitochondrial genomes are much larger and contain a significant amount of non-coding DNA [18]. However, the majority of mitochondrial genome sequencing work is primarily focused on animal mitochondria, rather than plant mitochondria, which hinders our understanding of the unique characteristics and functions of plant mitochondrial genomes.

Within Orchidaceae, only ten mitochondrial genome sequences have been published, covering three subfamilies: Epidendroideae, Cypripedioideae, and Apostasioideae. There are significant differences in the mitochondrial genome sizes among orchid plants, with the largest and smallest sizes both found within Epidendroideae. Specifically, *G. elata* has the largest mitochondrial genome size of 1,339,825 bp [19], while *Epipogium roseum* has the smallest size of 414,552 bp [20]. In this study, we present the first report on the mitochondrial genome of *A. fujianica*, belonging to Apostasioideae, with a size of 573,612 bp and a GC content of 44.5%. Notably, its size closely resembles that of *P. aphrodite* from Epidendroideae (576,203 bp) [21]. However, the similarity in mitogenome size does not necessarily imply a high degree of similarity in mitochondrial genes between them (Table 2). In terms of annotated gene count, as a primitive orchid [16], *A. fujianica* exhibits the fewest annotated genes (30), while *P. aphrodite* boasts 38 conservative PCGs. Furthermore, within Cypripedioideae, the mitochondrial genome of *P. micranthum* encodes 39 out of the 41 protein-coding genes present in the common ancestor of angiosperms, making it the most abundant and conservative mitogenome among orchids [22]. Despite this, its mitochondrial genome length is notably smaller (447,368 bp) compared to other orchid species. We speculate that this occurrence may be attributed to a loss or reduction in the length of non-coding regions. Additionally, it is evident that the *Dendrobium* genus possesses a notably high number of tRNA genes, typically three to four times as many as in other orchid species. However, the sequence length of these tRNA genes constitutes a negligible proportion of the entire genome (less than 1%). Hence, the significant expansion of the mitochondrial genome in *Dendrobium* is likely associated with non-coding region sequences. In our results, we also found significant size differences between two species of the same *Gastrodia* genera. This phenomenon has also been observed in other plant taxa, such as genera *Viscum* [23,24] and *Silene* [6,25]. The reasons for such size variations in close species may be related to repeated sequences and foreign sequences [26]. Nevertheless, the mechanism behind this expansion in non-coding regions and its potential correlation with foreign gene fragments (plastid or nuclear genes) remains unclear, requiring further exploration in future studies.

In many plants, the loss and transfer of mitochondrial genes is an ongoing process, which is why there are significant differences in the mitochondrial gene content among species [27]. Ancestral angiosperms possessed a complete set of 42 protein-coding genes in their mitogenomes. However, due to occasional functional transfers to the nucleus/chloroplast, most higher plants exhibit varying numbers of protein-coding genes, ranging from 24 to 42, along with two to three rRNA genes, with significant conservation differences among these genes [28,29]. From Table 2, it can be observed that *P. micranthum* retains the most complete set of mitochondrial genes (39) among the orchid species. In addition, the rRNA count in *Apostasia* lacks *rrn26*, which encodes 26s RNA [30]. Furthermore, *G. pubilabiata* and *P. aphrodite* have only nine tRNA genes, whereas *Dendrobium* species has over 30 tRNA genes, with approximately 25% of them being chloroplast-derived tRNAs. We believe that intergenomic transfer (IGT) is the main contributor to the high tRNA count in *Dendrobium* [31]. In the mitochondrial genome of angiosperms, the *sdh3* and *sdh4* genes are often simultaneously absent. The mitogenome of Fabaceae lacks the *cox2* gene, while in dicotyledonous plants, the *rps2* and *rps11* genes are largely lost [32,33]. From our observation of the phylogenetic tree, orchids primarily lose the *rpl10*, *rpl14*, *sdh3*, and *sdh4* genes (except for the *sdh4* gene in *P. micranthum*), while the *rps10* and *rps11* genes are not lost in orchids. These features may serve as molecular markers to distinguish orchids from other monocots. Additionally, the loss of these genes may indicate that orchids have made adjustments in their protein synthesis and energy metabolism pathways [21]. Orchids might compensate for the absence of these genes through alternative pathways or mechanisms, thereby gaining a survival advantage in specific environments. Additionally, in the orchid family, we found that *Apostasia* lacks the *cox2*, *nad1*, *nad2*, *nad4*, and *rps2* genes, while *Dendrobium* lacks the *nad5* gene. Recent studies on mitochondrial genomes have demonstrated the significance of intracellular gene transfer (IGT) in genetic material exchange, which play crucial roles in the evolution of plant mitochondrial genomes [34]. Additionally, we have found that the majority of plastid tRNA genes in *A. fujianica* have migrated to the mitochondrial genome. Among them, *trnN*-*GUU*, *trnD*-*GUC*, *trnW*-*CCA*, *trnP*-*UGG*, and *psaJ* have been identified as originating from chloroplast sequences. These transfers appear to be crucial for mitochondrial gene translation [35].

Due to slow nucleotide substitution rates in coding genes, mitochondrial genes are frequently utilized in phylogenetic analyses, particularly for reconstructing ancient phylogenetic relationships [3,36,37]. Based on 28 protein-coding genes (PCGs), we reconstructed a mitochondrial phylogenetic tree of monocotyledonous plants using the maximum likelihood (ML) method. The classification of Orchidaceae in this tree is largely consistent with classifications based on plastid and nuclear genomes, represented as follows: ((*A. fujianica*, *A. shenzhenica*) (*P. micranthum* ((*G. elata*, *G. pubilabiata*) (*P. aphrodite* (*D. henanense*, *D. wilsonii*))))). However, our classification of *Oryza*, based on 28 PCGs, contradicts the phylogenetic tree constructed using the neighbor-joining (NJ) method, based on 20 mitochondrial PCGs. We support a closer relationship between *O. sativa* and *O. rufipogon* [38] and advocate for mitochondrial genomics as a biological tool for resolving phylogenetic relationships at the level of plant families, orders, or higher taxa. In the progress of biological evolution, repetitive sequences are important products of genetic material exchange and recombination [39]. It is well known that plant mitochondrial genomes contain a large number of non-tandem repeat sequences, which can be used for genetic and evolutionary studies [40]. In this study, we identified long repeats and SSRs of the *A. fujianica* mitogenome. We found that long repeats of 30–39 in *A. fujianica* are the most abundant (250–300), a similar situation to *A. shenzhenica* (367–392) [12]. In SSR analysis, the proportion of A/T repeats is highest among all SSRs, a pattern that has also been observed in *E. roseum* [20]. Our selection pressure analysis found that the Ka/Ks value of *nad6* is greater than 1 (1.35), indicating that this gene is undergoing positive selection. Mutations in the *nad6* gene are more likely to change its encoded protein, and these changes are likely to provide some survival or adaptive advantage, possibly including improved energy production efficiency, the ability to adapt to environmental changes, or other traits related to mitochondrial function. As a result, these advantageous mutations have been naturally selected and retained in orchids. Additionally, RNA editing is a deamination reaction crucial for gene expression in the mitochondrial genome of higher plants. Investigating RNA modification target sites further deepens our understanding of the molecular mechanisms of gene expression in plant mitogenomes [41]. Through the analysis of RNA editing sites in the mitochondrial genomes of eight orchid species, we observed significant differences in the number of RNA editing sites among these species. For example, *A. fujianica* has the fewest RNA editing sites (287), while *G. elata* has the highest number, reaching 508. Additionally, the nad4 gene has the highest number of RNA editing sites (around 50 sites) across all studied orchid species, indicating its crucial role in mitochondrial function and expression. These findings suggest that RNA editing plays a key role in the variability and adaptive evolution among orchid species, reflecting the mechanisms by which orchid mitochondrial genomes adapt to environmental changes and ecological pressures through RNA editing. These results not only enhance our understanding of the diversity and adaptive evolution of orchid mitochondrial genomes but also provide a theoretical basis for the conservation and breeding strategies of orchids.

## 4. Materials and Methods

### 4.1. Plant Material, DNA Extraction and Sequencing

The plant material *A. fujianica* was collected from the Key Laboratory of National Forestry and Grassland Administration for Orchid Conservation and Utilization, Fujian Agriculture and Forestry University, China (119°13′ E, 26°05′ N). Total genomic DNA was extracted from fresh leaves using the Plant Genomic DNA Kit (Tiangen, Beijing). Then, the platforms Illumina NovaSeq (San Diego, CA, USA) and Pacbio Sequel (Pacific Biosciences, Menlo Park, CA, USA) were used to sequence short read and long read, respectively. We used SOAPnuke1.5.6 (https://github.com/BGI-flexlab/SOAPnuke, accessed on 16 October 2023) to remove adaptor and low-quality data (bases with quality value Q ≤ 20 that account for more than 10% of the entire read and the proportion of ‘N’ greater than 1%), following the following specific parameters: -n 0.01 -l 20 -q 0.1 -i -Q 2 -G -M 2 -A 0.5 -d.

### 4.2. Assembly, Annotation and Condon Usage Analysis

SPAdes v3.10.1 [42] was employed for de novo assembly of the mitogenome of *A. fujianica*, with parameters Kmer = 77, 101, and 127. In order to further confirm the assembly quality, software Quality Assessment Tool for Genome Assemblies (QUAST) v5.2.0 [43] was utilized to assess the quality under these three different Kmer values, and we selected the optimal assembly with Kmer = 127 for the mitochondrial genome. Then, we manually identified and selected a final mitochondrial scaffold, and designed 25–30 bp primers based on the flanking sequences of assembled contigs for PCR amplification, which could fill the gaps in the scaffold to obtain a complete mitogenome.

With the closely related species *A. shenzhenica*, selected as the reference sequence, Geneious Prime v2023.2.1 [44] was used to predict and annotate the coding protein genes, rRNA, and tRNA of the assembled mitogenome. Subsequently, through manual verification and correction, we ensured the accuracy of the annotation results. Finally, OGDRAW version 1.3.1 [45] was performed for visualization, illustrating the types and quantities of mitochondrial genes, as well as the GC content of *A. fujianica* mitogenome.

CodonW v1.4.4 (http://codonw.sourceforge.net/, accessed on 29 October 2023) was used to analyze relative synonymous codon use (RSCU) for *A. fujianica* mitogenome. Plotting was conducted using the “Organelle Analysis” tool on the GenePioneer Biotechnology Cloud Platform (http://cloud.genepioneer.com, accessed on 24 December 2023).

### 4.3. Repeat Sequence and Selective Pressure Analysis

To identify long sequence repeats (LSRs) of *A. fujianica*’s mitogenome, the online website REPuter (https://bibiserv.cebitec.uni-bielefeld.de/reputer, accessed on 25 December 2023) was employed to detect forward (F), palindrome (P), reverse (R), and complement (C) types, using the following parameters: a maximum size of 50, a minimum size of 20, and a hamming distance of 3. Additionally, the MISA Perl script (https://webblast.ipk-gatersleben.de/misa/index.php?action=1, accessed on 27 December 2023) was used to identify simple sequence repeats (SSRs). And in the MISA configuration settings, the definition parameter was adjusted to 1-10 2-5 3-4 4-3 5-3 6-3.

To analyze the selective pressure of 26 protein-coding genes (PCGs) between eight orchid species (*A. fujianica*, *A. shenzhenica*, *D. wilsonii*, *D. henanense*, *G. pubilabiata*, *G. elata*, *P. aphrodite*, and *P. micranthum*), DnaSP 6 v6.12.03 [46] was utilized to calculate the Ka, Ks, and Ka/Ks values, with the following parameter adjustments: the genetic code was set to nuclear universal, and the protein-coding regions were maintained at default settings. In addition, the box plot was generated using the BioLadder Bioinformatics Cloud Platform (https://www.bioladder.cn, accessed on 10 January 2024).

### 4.4. Gene Transfer, RNA Editing Sites Prediction, and Phylogenetic Analysis

To analyze the sequence similarity between the chloroplast [47] and mitochondrial genome of *A. fujianica*, we conducted sequence alignment of two organelle genomes using BLASTN [48], with an e-value cut-off set to 1 × 10^−5^. For better visualization, we utilized the Advanced Circus function of TBtools v2.096 [49] for plotting.

We performed RNA editing site prediction for 28 protein-coding genes (28 PCGs) across eight species of Orchidaceae, including the previously mentioned six, as well as *A. shenzhenica* and *D. henanense*. The prediction of RNA editing sites for *A. fujianica’s* mitogenome was conducted using the Organelle Analysis module on the GenePioneer Biotechnology Cloud Platform (http://cloud.genepioneer.com, accessed on 21 January 2024).

To construct a maximum likelihood (ML) phylogenetic tree, we downloaded mitochondrial genome data of 21 plant species from the National Center for Biotechnology Information (NCBI, https://www.ncbi.nlm.nih.gov/, accessed on 20 February 2024), including two dicotyledonous plants, *Gossypium harknessii* and *G. hirsutum*, selected as outgroups. Initially, based on our previous method, we exported mitogenome annotations of 22 species and extracted 28 core PCGs from each annotation using Geneious Prime v2023.2.1 [44]. Then, we aligned the extracted 28 FASTA model files using MAFFT-v7.409 [50], concatenated the sequences, and used the PartitionFinder module in PhyloSuite v1.2.2 [51] to generate partition mode. Next, we constructed the ML phylogenetic tree using IQ-TREE with 1000 bootstrap replicates. Finally, we visualized the ML tree using FigTree v1.4.4 and simultaneously displayed the distribution of 42 PCGs across the mitogenomes of 22 species.

## 5. Conclusions

Our study is the first to reveal the complete mitogenome of *A. fujianica*, with a total length of 573,612 bp and a GC content of 44.5%. Our results show that *A. fujianica* has the fewest mitochondrial genes among the currently known orchid species, and that the number of tRNAs in the genus *Apostasia* is one fewer than in other orchids (missing *rrn26*). The *nad6* gene was found to be under positive selection. In the process of the IGT event of *A. fujianica’s* mitogenome, the *trnN*-*GUU*, *trnD*-*GUC*, *trnW*-*CCA*, *trnP*-*UGG*, and *psaJ* genes were identified as having transferred from plastid to mitochondrion. Through phylogenetic analysis of the mitochondrial genomes of *A. fujianica* and 19 other monocot species, the evolutionary relationships of the monocots were determined. Furthermore, compared to other monocots, Orchidaceae primarily lost the *rpl10*, *rpl14*, *sdh3*, and *sdh4* genes. Based on these findings, we believe our results provide valuable supplementary mitochondrial data for the Orchidaceae, enhancing our understanding of their evolutionary relationships and genetic diversity.

## Figures and Tables

**Table 1:** Characteristics of the complete mitogenome of *A. fujianica*. * Genes were incomplete or showed pseudogenization.

Contig	Length (bp)	GC Content (%)	Genes
mt1	120	37.5	/
mt2	22,467	42.9	*nad6*
mt3	3110	49.8	/
mt4	116	44.8	*nad3* *
mt5	382	43.5	*nad3* *
mt6	188	47.3	/
mt7	12,321	42.0	*cob*, *rps14*, *rpl15*
mt8	20,753	44.4	*trnQ*-*UUG*, *rps12*
mt9	38,670	45.4	*trnK*-*UUU*, *ccmC*
mt10	23,805	44.9	/
mt11	7824	43.9	/
mt12	37,110	44.1	*atp9*, *cox3*, *rrn5*, *rrn18*
mt13	38,175	46.2	*nad7*, *trnE*-*UUC*
mt14	164,718	44.3	*nad5*, *trnC*-*GCA*, *cox1*, *ccmFc*
mt15	37,436	44.7	*matR*, *tRNA*-*Cys*
mt16	6018	47.7	*trnN*-*GUU*
mt17	3434	44.5	/
mt18	34,527	45.0	*rps10*
mt19	25,307	45.8	*nad4L*, *atp4*, *trnW*-*CCA*, *trnP*-*UGG*, *psaJ*, *rpl16*, *rps3*, *rps19*, *rpl2*
mt20	10,861	41.8	*tRNA*-*Met*, *ccmFn*, *atp1*
mt21	25,659	44.5	*rps11*, *trnQ*-*UUG*, *rps13*
mt22	16,559	43.0	*trnD*-*GUC*, *atp8*
mt23	5887	44.9	*nad9*, *trnY*-*GUA*
mt24	10,780	43.6	*rps4*
mt25	27,385	44.1	*rps7*

**Table 2:** Features of orchid mitogenomes.

Feature	*A. fujianica*	*A. shenzhenica*	*D. henanense*	*D. wilsonii*	*G. elata*	*G. pubilabiata*	*P. micranthum*	*P. aphrodite*
Genome size (bp)	573,612	672,872	807,551	763,005	1,339,825	867,349	447,368	576,203
GC content (%)	44.5	44.4	43.1	43.7	44.6	42.1	44.4	44.3
Number of PCGs	30	36	38	38	37	38	39	38
Number of tRNA	12	16	40	33	20	9	16	9
Number of rRNA	2	2	3	3	3	3	3	3
Total genes	44	54	81	74	60	50	58	50

## Data Availability

Data are contained within the article and Supplementary Table S2. The newly completed mitogenome accession numbers: PP724664 (*Apostasia fujianica*).

## Supplementary Materials

The supporting information can be downloaded at: https://www.mdpi.com/article/10.3390/ijms25158151/s1.
